# Water-Soluble Polysaccharides from *Ephedra alata* Stems: Structural Characterization, Functional Properties, and Antioxidant Activity

**DOI:** 10.3390/molecules25092210

**Published:** 2020-05-08

**Authors:** Leila Soua, Mohamed Koubaa, Francisco J. Barba, Jawhar Fakhfakh, Hanen Kolsi Ghamgui, Semia Ellouz Chaabouni

**Affiliations:** 1 Laboratory for the Improvement of Plants and Valorization of Agroresources, National School of Engineering of Sfax (ENIS), University of Sfax, Sfax 3038, Tunisia; leilasoua115@gmail.com (L.S.); kolsighamgui.hanen@yahoo.fr (H.K.G.); semia.chaabouni@enis.rnu.tn (S.E.C.); 2 ESCOM, UTC, EA 4297 TIMR, 1 Allée du Réseau Jean-Marie Buckmaster, 60200 Compiègne, France; m.koubaa@escom.fr; 3 Nutrition and Food Science Area, Preventive Medicine and Public Health, Food Science, Toxicology and Forensic Medicine Department, Universitat de València, Faculty of Pharmacy, Avda; Vicent Andrés Estellés, s/n, 46100 Burjassot, València, Spain; 4 Laboratory of Organic Chemistry, Natural Substances section, Faculty of Sciences of Sfax, PB 802, Sfax 3018, Tunisia; jawharfakhfakh@yahoo.fr

**Keywords:** *Ephedra alata*, polysaccharides, physicochemical characterization, functional properties, antioxidant activities

## Abstract

In this study, the physicochemical characterization, functional properties, and antioxidant activity of polysaccharides extracted from *Ephedra alata* (EAP) were investigated. EAP were extracted in water during 3 h with a liquid/solid ratio of 5 in a water bath at 90 °C. The structure of the extracted EAP was examined by Fourier transform infrared spectroscopy (FT-IR), scanning electron microscopy (SEM), and gas chromatography-mass spectrometry (GC-MS). The functional properties and biochemical activities of EAP were determined. The chemical analysis revealed that the contents of carbohydrates, uronic acid, and proteins were 73.24% ± 1.24%, 6.82% ± 0.57%, and 6.56% ± 0.36%, respectively. The results showed that the extracted EAP essentially contain three functional groups: C=O, C-H, and O-H. SEM images showed that EAP present numerous high porosity particles. The monosaccharide composition revealed a polymer composed of glucose (43.1%), galactose (36.4%), mannose (14.9%), arabinose (3.7%), and gluconic acid (1.7%). EAP showed interesting functional properties (solubility, oil holding capacity, foaming and emulsion properties). Finally, the results revealed that EAP displayed excellent antihypertensive and antioxidant activities. Overall, EAP present a promising natural source of food additives, antioxidants, and antihypertensive agents.

## 1. Introduction

Polysaccharides are carbohydrate polymers that are widely present in plants, animals, and microorganisms [1]. They have drawn considerable attention owing to their widespread use in different industrial fields such as foods and pharmaceuticals [2]. Many research works have focused on their functional properties (e.g., water holding capacity, oil holding capacity) [3] and biological activities (e.g., anticancer, antioxidant, antitumor, immunomodulatory, etc.) [4,5]. It has been demonstrated that the functional properties of polysaccharides are firmly dependent on their basic structure, which includes their molecular weight, monosaccharides composition, and the configuration of the glycosidic bonds [6,7].

*Ephedra alata*, commonly known in Tunisia as ″alenda″, belongs to the Ephedraceae family containing more than 60 species of nonflowering seed plants, with light green densely branched dioecious small and perennial stiff shrub about 50–100 cm tall [8]. This species is mainly distributed in arid environments, often near shifting sand dunes of Iran, Algeria, Iraq, Chad, Egypt, Palestine, Lebanon, Jordan, Saudi Arabia, Morocco, Syria, Libya, Mauritania, Mali, Somalia, and Tunisia, where it grows wildly on the gravely rocky, sandy, and clay soil [8]. The decoction of *E. alata* stem is used in folk medicine as a potential stimulant, to treat different disorders (e.g., kidney, bronchi, circular system, digestive system disorders), to relieve asthma attack, and as antifungal [9]. The plant stems are usually chewed to treat bacterial and fungal infections [10].

The review of the literature shows that most of the studies of *E. alata* were focused on its content in alkaloids and phenolic compounds. Nonetheless, in other species of *Ephedra*, such as *E. sinica*, the extracted polysaccharides have proven to possess biological activities [11]. For instance, it has been demonstrated that they could be used to alleviate the airway and pulmonary inflammation and could become a new therapeutic drug for the treatment of chronic obstructive pulmonary diseases (COPD) [11]. Acidic polysaccharides extracted from *E. sinica* were found to possess immunosuppressive activity and it was demonstrated that the main acidic polysaccharide, ESP-B4, had potential therapeutic effects on rheumatoid arthritis. ESP-B4 reduces the release of inflammatory factors and cytokines by inhibiting the toll like receptor 4 (TLR4) signaling pathway [12]. In addition, it was found that, in the *Ephedra* root extracts, ephedrannin A (72) and ephedrannin B (73) had anti-inflammatory effects. They could suppress the transcription of the tumor necrosis factor (TNF-α) and Interleukin (IL-1β) and inhibit the lipopolysaccharide-induced inflammation [13]. In another study, it was found that the methanolic extracts of *E. major* aerial parts and roots inhibited the fungal growth and the production of aflatoxin B1 (AFB1), dose-dependently [14]. Moreover, the essential oil extracts from the aerial parts of this plant significantly inhibited the fungal growth at the highest concentration of 1000 μg·mL^−1^ without any obvious effect on AFB1 production at all concentrations tested (0–1000 μg·mL^−1^) [14].

Angiotensin-converting enzyme (ACE) is a dipeptidyl carboxypeptidase (EC 3.4.15.1) and is widely distributed in mammalian tissues [15]. It converts angiotensin I to angiotensin II, a vasoconstrictor. It also inactivates bradykinin, which is a vasodilator peptide. This enzyme plays an important role in the regulation of blood pressure through these two mechanisms [16]. ACE inhibitors are effective antihypertensive agents. It is thus interesting to investigate how ACE improves the body′s antioxidant capacity for health benefits. Furthermore, in the conditions of hypertension, angiotensin II amplifies the oxidative stress as it disturbs many of its cellular functions through stimulating the formation of intracellular reactive radical species (ROS) [17]. Therefore, in addition to blood pressure control, ACE inhibitors have been shown to intensify the antioxidant defense system in animals and humans by inhibiting the formation of angiotensin II [18].

Besides, studies on the antioxidant potential of polysaccharides isolated from plant species have increased remarkably owing to their strong efficiency [19]. Moreover, as most of the synthetic antioxidants are potentially harmful to humans, it is particularly important to seek natural antioxidants instead. In fact, most of the polysaccharides extracted from natural resources present low toxicity and exhibit a strong biological activity when compared with other natural antioxidants [20]. It should be noted that many plant-based products rich in polysaccharides, such as that from *Astragalus membranaceus*, exist in the market and are commercialized for their health-related properties.

Owing to the above-mentioned beneficial properties of polysaccharides, and especially those extracted from *Ephedra* species, the current work aims to extract water-soluble polysaccharides from *E. alata* (EAP) stems, and then to study their antioxidant activity, functional properties, as well as physicochemical characterization.

## 2. Results and Discussion

### 2.1. EAP Content and Chemical Composition

The recovered EAP content was 4% ± 1.02%, which is higher than that obtained from *E. sinica* stems (0.85%) [21], and in the same range as that extracted from *E. sinica* Stapf stems (4.9%) [22]. Such variability in the extraction yields could be related to several factors such as environmental conditions, habitat, physiological factors, growth cycle, and seasonal variations [23]. The harvesting period could be a major factor responsible for the low extraction yield. Besides, it was reported that the extraction yield of polysaccharides is highly influenced by the extraction time and temperature, as well as the solid/liquid ratio [24]. The chemical composition of the extracted EAP on a dry weight basis (Table 1) shows that the carbohydrates present the most important part (73.24% ± 1.94%). This value was higher than that reported for water-soluble polysaccharides extracted from *Carex meyeriana* Kunth, which was about 38.28% [25]. Moreover, the uronic acid content was about 6.82% ± 0.57%, which was higher than that found in the polysaccharides extracted from *C. meyeriana* Kunth (4.76% ± 0.48%) [25]. Low protein content (6.56%) was present in the extracted EAP, which was similar to that reported by Hu et al. [25] for polysaccharides extracted by hot water from *C. meyeriana* Kunth (6.38% ± 0.28%). It should be noted that the extraction yield of polysaccharides is highly influenced by the extraction time and temperature, as well as the solid/liquid ratio [24].

The ash content of EAP was about 10.24% ± 0.24%, which was higher than some commercially available polysaccharides, such as agar and carrageenan, ranging from 2.5% ± 0.03% to 2.7% ± 0.031% [26]. Regarding the mineral composition of EAP extract, the results presented in Table 2 show that sodium was the most abundant mineral (223.2 mg/100 g), followed by calcium (197.85 mg/100 g), and then potassium (108.5 mg/100 g). As reported by Jayasinghe et al. [26], the commercial agar contains a similar amount of Mg and K, whereas EAP present high levels of Na and Ca.

The measurement of Aw shows that the EPA present an Aw of 0.37 ± 0.006, which is situated according to the map of stability in an area where the oxidation of lipids is low, non-enzymatic browning (Maillard reaction) is slow, and the enzyme activities are required to prevent the development of microorganisms. When polysaccharides are used in food formulations, they should not give any off-flavor or produce any side effects in the final products. Moreover, the EAP showed a high light value (L* = 70.09 ± 0.08). The a* and b* values recorded were 6.05 ± 0.01 and 20.23 ± 0.03, respectively (Table 1). These results concur with previous ones reported by Ben Jeddou et al. [27], showing that the polysaccharides extracted from potato peels displayed light color and a slight degree of redness.

### 2.2. UV/Visible Spectroscopy

**Figure 1:**
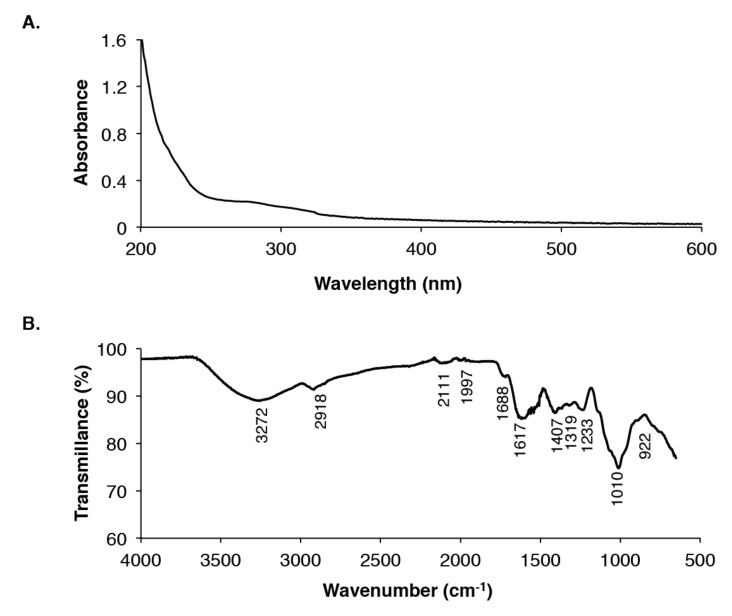
(**A**) UV absorption spectrum of *Ephedra alata* (EAP). (**B**) Fourier transform infrared (FT-IR) spectrum of EAP.

The maximum absorption peaks of EAP found in the UV/visible spectrum ranged from 200 to 240 nm (Figure 1A). On the basis of their absorption maxima between 204 and 216 nm and the absence of any absorption in the range of 260–280 nm, EAP was identified as polysaccharides and not proteins or nucleic acids [2].

### 2.3. Monosaccharide Composition

The monosaccharide composition of EAP determined by gas chromatography-mass spectrometry (GC-MS) revealed the presence of different carbohydrate moieties in varied proportions (Table 3). Acid hydrolysis of EAP showed that glucose was the most abundant sugar (43.1%), followed by galactose (36.4%), mannose (14.9%), arabinose (3.7%), and gluconic acid (1.7%).

### 2.4. Fourier Transform Infrared (FT-IR) Spectroscopy Analysis of EAP

FT-IR spectroscopy was performed in the region ranging from 4000 to 400 cm^−1^ to further characterize the EAP. As presented in Figure 1B, EAP displayed typical peaks of polysaccharides at 3272, 2918, 1617, 1407, 1233, 1010, and 922 cm^−1^. The peak observed at 3272 cm^−1^ corresponds to the stretching vibration of OH groups owing to inter- and intra-molecular hydrogen bands [28]. The peak observed at 2918 cm^−1^ represents the stretching of CH groups of the free sugars [29]. Furthermore, the peaks at 1617 cm^−1^ and 1688 cm^−1^ were attributed to carbonyl ester (C=O) groups [30], whereas the region between 1617 cm^−1^ and 1688 cm^−1^ reflects the bound water [31]. Finally, the region between 922 cm^−1^ and 1010 cm^−1^ is attributed to the presence of carbohydrate fingerprints for the functional groups characterizing polysaccharides as bending (O-H), stretching (C-O-C), and deforming (CH_3_) vibrations. The peaks below 1000 cm^−1^ also indicate the presence of visible bands and/or the possible linkages between two monosaccharide molecules [24].

### 2.5. Scanning Electron Microscopy (SEM)

**Figure 2:**
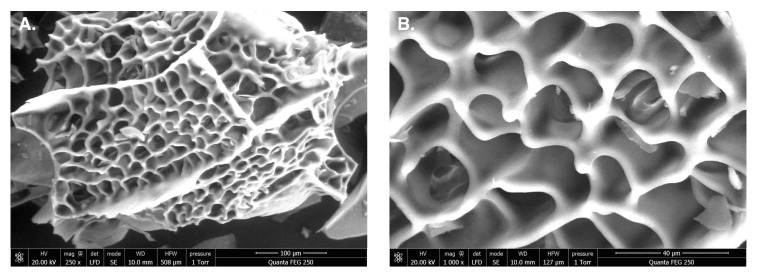
Scanning electron microscopy images of EAP. (**A**). Image magnified 250 times; (**B**). Image magnified 1000 times.

The shape and surface characteristics of the EAP were investigated using SEM analysis and the obtained images are presented in Figure 2A,B. The results showed that the EAP present numerous large size and high-porosity particles. These results were different from those obtained by Cheng et al. [32], in which it was reported that *E. acuminatum* polysaccharides had a flat and smooth surface.

### 2.6. Functional Properties

#### 2.6.1. Water-Holding (WHCs) and Fat-Binding Capacities (FBCs)

WHC and FBC are functional properties in food processing that are closely related to texture via the interaction between the components, including water and oil. The results show that EAP have a WHC of 2 ± 0.10 g H_2_O/g sample, which is higher than commercial citrus pectin (1.38 ± 0.06 g H_2_O/g sample), Arabic gum (0.28 ± 0.15), and wheat starch (0.74 ± 0.02), previously reported by Gannasin et al. [33]. The FBC exhibited by EAP (4 ± 0.1 g oil/g sample) was also higher than that displayed by commercial citrus pectin (1.55 ± 0.09 g oil/g sample), Arabic gum (1.00 ± 0.10 g oil/g sample), and wheat starch (0.92 ± 0.03 g oil/g sample). This difference is probably linked to the porosity of EAP structure rather than the affinity of the polysaccharide to oil [34].

#### 2.6.2. Foaming Properties

**Figure 3:**
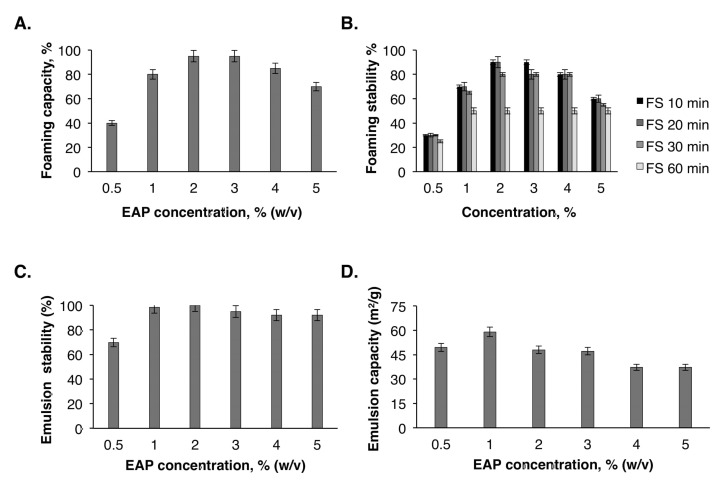
(**A**) Foam capacity of EAP. (**B**) Foam stability (FS) of EAP. (**C**) Emulsion stability of EAP. (**D**) Emulsion capacity of EAP.

Foam development and stability largely depend on the interfacial properties of the surface-active components used in the formulation [35]. Foam capacity (FC) and foam stability (FS) of EAP were assessed at different concentrations (0.5%, 1%, 2%, 3%, 4%, and 5% *w*/*v*). The results obtained (Figure 3A) show a high FC of EAP at 2% and 3% concentrations. In contrast, the FC decreased slightly from 95% ± 2.78% to 85% ± 1.41% at a concentration of 4%. The foaming capacity of EAP was better than some commercial hydrocolloids such as bovine gelatin (61.92%) and Arabic gum (25%) [33]. Moreover, carrageenan, wheat starch, and xanthan gum have no FC [33]. On the other hand, the foam made in the presence of 2% EAP was stable and decreased slightly after 60 min (Figure 3B). These interesting FC and FS values of the EAP suggest their capacity to increase the viscosity of various food formulations and forming a system stabilizing the interfacial gas–liquid film [36].

#### 2.6.3. Emulsion Capacity (EC)

An emulsion is an intimate mixture of two or more liquids that are generally immiscible. The study of the EC and emulsion stability (ES) of EAP at various concentrations (0.5%–5% *w*/*v*) (Figure 3C,D) shows that the highest EC value was obtained at a concentration of 1%. From a concentration of 3%, the EC started to decrease gradually until 37.18 m^2^/g at 5%. Concerning the ES, the formed emulsions were stable at a concentration of 2% and then decreased below this concentration, which was equal to 92% at a concentration of 5%. A similar trend was observed by Benhura et al. [37], who evaluated the ES of Arabic gum at concentrations ranging from 0.25% to 1%. The obtained results in the current work show that the efficiency of EAP depends on the concentration of this polymer in the aqueous phase of the emulsion.

### 2.7. Antioxidant Activities of EAP

#### 2.7.1. Determination of Total Antioxidant Capacity (TAC)

**Figure 4:**
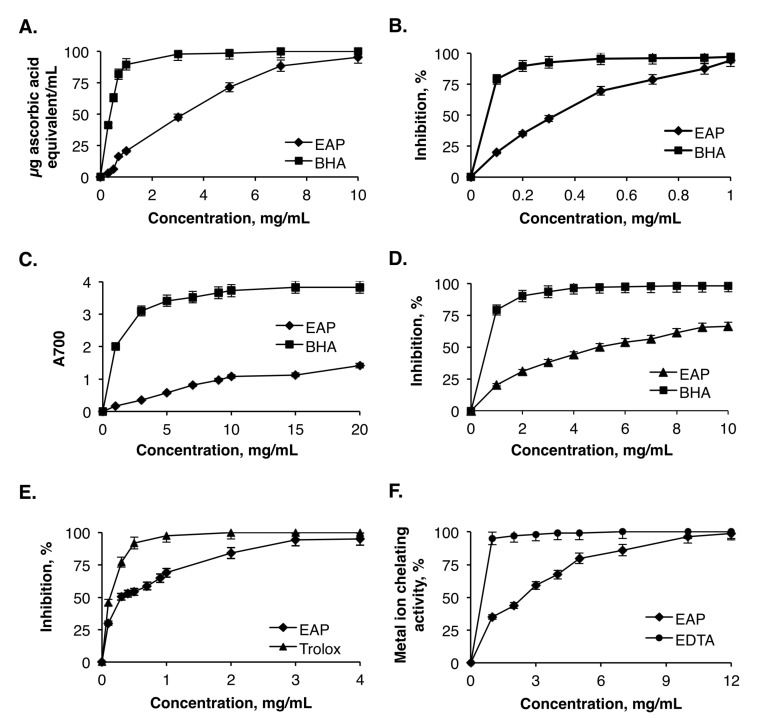
(**A**) Total antioxidant capacity of EAP. (**B**) DPPH (2,2-diphenyl-1-picrylhydrazyl) free radical scavenging activity of EAP. (**C**) Reducing power capacity of EAP. (**D**) β-Carotene bleaching activity of EAP. (**E**) ABTS (2,2’-azino-bis(3-ethylbenzothiazoline-6-sulfonic acid) free radical-scavenging activity of EAP. (**F**) Ferrous ion-chelating activity of EAP. BHA, butylated-hydroxyanisole.

The phosphomolybdate method is based on the reduction of Mo (VI) to Mo (V) at acidic pH and the formation of a green molybdate phosphate complex in the presence of an antioxidant molecule. The results presented in Figure 4A indicate that the TAC was proportional to the concentration of EAP. The half maximal inhibitory concentration (IC_50_) of EAP was estimated to be 3.2 mg/mL. This value was higher than that obtained for butylated-hydroxyanisole (BHA) (IC_50_ = 0.4 mg/mL) used as the positive control. Despite the lower antioxidant activity compared with that of BHA, the obtained results indicated that EAP present an interesting natural antioxidant extract [26].

#### 2.7.2. DPPH Free Radical Scavenging Activity

As a stable free radical, DPPH has been widely used to evaluate the free radical scavenging capacity of natural compounds by donating hydrogen to form a stable DPPH molecule [38]. The free radical scavenging activity of EAP was determined and compared to the reference antioxidant BHA. Figure 4B describes the DPPH scavenging ability of EAP, and shows an obvious antioxidant activity in a concentration-dependent manner. For all concentrations, EAP showed a dose-dependent DPPH radical scavenging activity, with an effective scavenger at lower concentrations (0.2–0.3 mg/mL). At 0.3 mg/mL, the DPPH scavenging activity of EAP was 47.32% ± 0.32%, which was slightly lower than the polysaccharide fraction reported by Chen et al. [39] (55.74% ± 4.02%). At a higher concentration range (0.5 to 1 mg/mL), the DPPH scavenging activities of EAP increased proportionally to the concentrations. The interesting EAP radical scavenging activity was probably owing to the presence of hydroxyl and carboxyl groups of uronic acids in the polysaccharides, which act as a hydrogen donor to scavenge the DPPH free radicals and, consequently, neutralize the effect of oxidative stress.

#### 2.7.3. Reducing Power Capacity

The reducing power capacity of EAP to convert Fe^3+^ to Fe^2+^ was followed by measuring the absorbance at 700 nm, corresponding to the formation of Perl′s Prussian blue at 700 nm. The results in Figure 4C show that the ferric reducing power increased proportionally to the concentrations of EAP within the range of 1–20 mg/mL. However, compared with BHA, EAP exhibited a relatively lower activity. It has been reported that the reducing power of plant polysaccharides was often associated with the capacity of reacting with given precursors of peroxide [40].

#### 2.7.4. β-Carotene Bleaching Assay

The β-carotene bleaching test simulates the oxidation of membrane lipids. It is thus considered a good model for membrane-based lipid peroxidation. The oxidation of linoleic acid during this assay generates peroxyl free radicals, which can oxidize the unsaturated β-carotene. The presence of an antioxidant molecule in the medium will minimize this oxidation. The antioxidant activity of EAP was assessed using the β-carotene/linoleic acid bleaching assay. As shown in Figure 4D, the trend of concentration dependence was clear. Considering the IC_50_ values, EAP (IC_50_ = 4.2 mg/mL) was found to be more efficient compared with the water-soluble polysaccharides extracted from almond by-product (IC_50_ = 4.46 mg/mL), but was lower than that of pistachio by-product (IC_50_ = 3.39 mg/mL) [41]. The IC_50_ value recorded for EAP was higher than that of BHA, which was in the range of 0.6 mg/mL.

#### 2.7.5. ABTS Free Radical-Scavenging Ability

The potential of EAP to scavenge free radicals was also assessed by their ability to quench ABTS^•+^. The results presented in Figure 4E show that the EAP exhibit a strong radical scavenging activity on ABTS at a concentration of 2 mg/mL and the removal of ABTS radical reached 84.17% ± 2.16%. At the same concentration, this value was higher than that reported by Hammami et al. [42] (72.32%). For instance, the scavenging effects of ABTS radicals on polysaccharides were relatively lower than that of Trolox at the same concentration. The kinetics of the antioxidant activity of the ABTS radical can be influenced by the chemical structure, the molecular weight, and the high sulfate level [42].

#### 2.7.6. Ferrous Ion-Chelating Activity

Chelating agents may inhibit lipid oxidation through disrupting the formation of Fe^2+^-ferrozine complex. In the current study, EAP exhibited an excellent chelating activity, which increased in a dose-dependent manner and reached 96.23% at the dose of 10 mg/mg. However, this activity was lower than that of ethylenediaminetetraacetic acid (EDTA) at all tested concentrations (Figure 4F). This chelating activity was much higher than that of laminaran purified from *Cystoseira barbata* seaweed, which was 78% at a concentration of 20 mg/mL [43]. The IC_50_ of Fe^2+^ ion chelating ability of EAP was equal to 2.4 mg/mL. These findings indicated that EAP can exhibit antioxidant activity by capturing iron ions. Song et al. [44] pointed out that the presence of carboxyl groups in galacturonic acid units significantly increases their chelating potentialities.

### 2.8. ACE Inhibitory Activity of EAP

**Figure 5:**
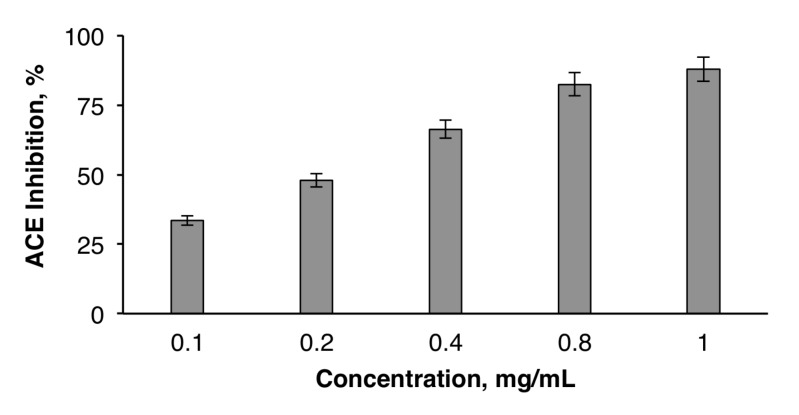
Angiotensin I-converting enzyme (ACE) inhibitory activity of EAP.

The inhibition of angiotensin I-converting enzyme (ACE) by dietary anti-hypertensive agents is a promising strategy for hypertension management. In fact, recent research showed that ACE inhibition might be a useful therapy for the treatment of high blood pressure. As synthetic ACE inhibitors may cause adverse side effects, plant molecules may offer natural and cost-effective alternative ACE inhibitors for the treatment and prevention of hypertension. The results presented in Figure 5 show that EAP exhibit ACE-inhibitory activity. This activity increased in a concentration-dependent manner. The ACE inhibitory effect of EAP (82.49% ± 0.66% at 1 mg/mL) was lower than that of *M. charantia* bioactive polysaccharide (94.1%) [45], but higher than that of polysaccharides extracted from almond by-product (79.5% ± 2.8% at 5 mg/mL) and pistachio by-product (81.78% ± 1.1% at 5 mg/mL) [41]. The IC_50_ value of EAP was also determined (IC_50_ = 0.21 mg/mL). It was equal to that of watermelon rinds (IC_50_ = 0.21 mg/mL), but ten times lower than that of polysaccharides derived from almonds (IC_50_ = 2.81 mg/mL) and pistachios (IC_50_ = 2.59 mg/mL) [41]. Although the ACE inhibitory mechanism of polysaccharides remains unknown, the inhibitory activity of EAP was presumed to be the result of the presence of galacturonic acid, which could be ionized when EAP was dissolved in water, and then the release of hydrogen ions. This process created an acidic environment, which was not suitable for ACE because its optimum pH is 8.3. Under acidic conditions, ACE would be denatured and thus lose its activity [22].

## 3. Materials and Methods

### 3.1. Plant Material

**Figure 6:**
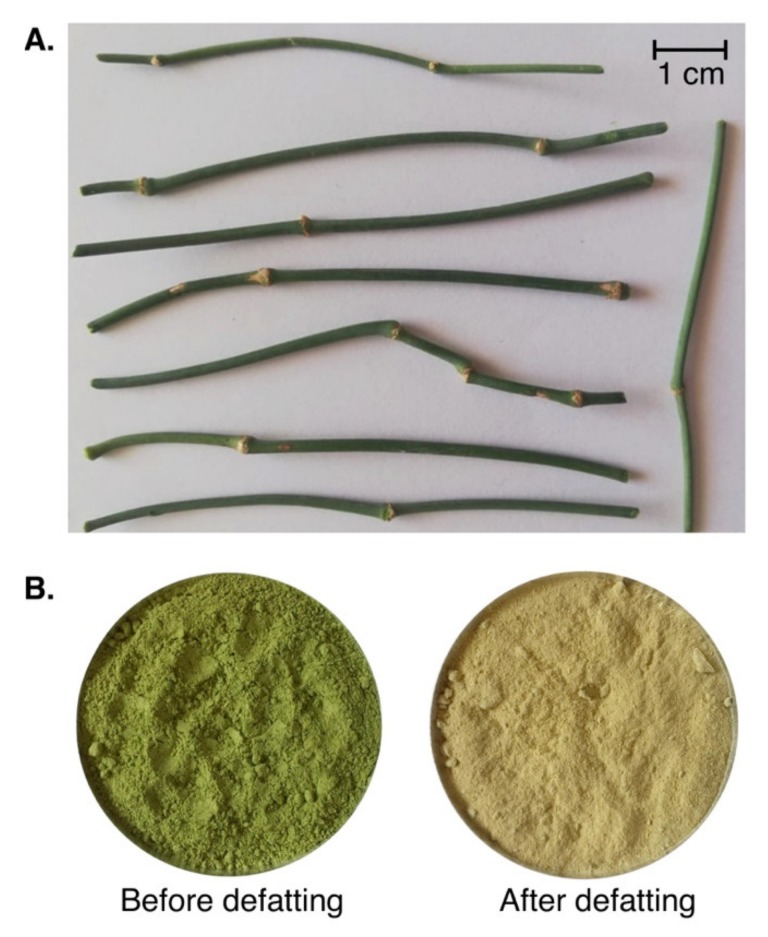
(**A**) Photo of *E. alata* stems. (**B**) *E. alata* powder aspects before and after defatting.

The stems of *E. alata* were collected in December 2017 from the region of Chebba (Mahdia city, Tunisia) (Figure 6A). The aerial parts were first washed with distilled water, dried at 50 °C in the oven for 48 h, and then ground to a fine powder in a mixer grinder (Moulinex, Fontenay-sous bois, France). The obtained powder was then stored at 4 °C until use.

### 3.2. Reagents

The chemicals obtained from Sigma Chemical Co. (St. Louis, MO, USA) were DPPH (2,2-diphenyl-1-picrylhydrazyl), angiotensin I-converting enzyme from rabbit lung, ACE synthetic substrate hippuryl-1-histidyl-1-leucine (HHL), butylated-hydroxyanisole (BHA), β-carotene, ascorbic acid, linoleic acid, and Tween 40. All the other chemicals, namely trichloro-acetic-acid (TCA), potassium ferricyanide, sodium hydroxide, and ferric chloride, were of analytical grade.

### 3.3. Extraction of Water-Soluble Polysaccharide

Water-soluble polysaccharides from *E. alata* stems (EAP) were extracted as previously described [12], with modifications. Dried aerial parts of *E. alata* were defatted with 95% ethanol solution for 2 h to eliminate small lipophilic molecules and impurities (Figure 6B). The defatted residue was mixed with distilled water for 3 h with a liquid/solid ratio of 5 in a water bath at 90 °C. The extraction step was performed twice to ensure the maximum yield of polysaccharides. The filtrates obtained were combined and concentrated 20-fold using a rotary evaporator maintained at 50 °C under vacuum. The extracted EAP were then precipitated by adding three volumes of absolute ethanol and incubation overnight at 4 °C. After centrifugation for 15 min at 5869× *g*, the pellet was solubilized in distilled water. The solution was dialyzed against deionized water to remove the inorganic salts, and then freeze-dried to obtain lyophilized polysaccharides. The extraction yield expressed in % was calculated as the ratio of the crude dried polysaccharides′ weight divided by that of the initial raw material and multiplied by 100.

### 3.4. Physicochemical Characterization of EAP

The total carbohydrate content of EAP was measured by the phenol-sulfuric acid method using glucose as standard [46]. Uronic acid was estimated by the carbazole method using glucuronic acid as standard for the calibration [47]. The total crude protein content of the samples was determined by Kjeldahl′ s method (N × 6.25) [48]. The moisture, ash, and lipid contents of EAP were analyzed according to the American Association of Cereal Chemists 2000 standard methods.

### 3.5. Monosaccharide Composition of EAP

The monosaccharide composition of the extracted EAP was performed by gas chromatography-mass spectrometry (GC-MS) after hydrolysis for 2 h with a trifluoroacetic acid (TFA) solution (2 M) at 120 °C. A sample of 50 mg lyophilized hydrolysate was silylated by a mixture of pyridine-hexamethyldisilazane-trimethylchlosilane, 9:3:1 (*v*/*v*/*v*). Analysis of the obtained derivatives from the trimethylsilyl sugars was carried out by a Varian 3800 chromatograph using a fused silica capillary column (30 m × 0.25 mm) coated with DB-225ms (Agilent Technologies, Les Ulis, France). The separation and elution conditions were as follows: the injected volume was 1 µL and the temperatures of the detector and the injector were both set at 320 °C. The temperature of the column was set at 100 °C for 1 min and then ramped from 100 to 260 °C at 4 °C/min, and held for 10 min at 260 °C. The carrier gas used was helium at a flow of 1 mL/min.

### 3.6. Water Activity of EAP

Water activity (A_w_) was measured using the apparatus A_w_ SPRINT TH-500 (Novasina, Lachen, Switzerland). The measurement consisted of filling a plastic capsule with EAP, followed by reading the A_w_ at 25 °C.

### 3.7. Color Determination of EAP

Color parameters of EAP powder were measured with a portable colorimeter Chroma Meter CR-410 (Konica Minolta Sensing, Osaka, Japan) based on L*, a*, and b* values. After calibration, the sample was placed on a holding device. L* value indicates the lightness, a* value gives the degree of the red-green color, and b* value indicates the degree of the yellow-blue color [49].

### 3.8. UV Absorption Spectrum of EAP

EAP was dissolved in distilled water to get a final concentration of 1 mg/mL, and analyzed for full band scanning in the range of 200–600 nm [27], using a UV spectrometer Helios Omega UV-Vis (Thermo Fisher Scientific, Villebon-sur-Yvette, France).

### 3.9. Infrared Spectroscopic Analysis of EAP

The Fourier transform infrared (FT-IR) spectrum of EAP was obtained using an FT-IR spectrophotometer Spectrum BX FT-IR (PerkinElmer, Villebon-sur-Yvette, France). The ground sample was incorporated into spectroscopic grade potassium bromide (KBr) powder and then pressed into a 1 mm disk. The results were recorded in the wavenumber range of 4000–400 cm^−1^.

### 3.10. Scanning Electron Microscopy of EAP

The EAP were first coated with gold using a sputter coater JFC-1100 (JEOL Ltd., Tokyo, Japan), and then examined by a scanning electron microscope JSM-5400 (JEOL Ltd., Tokyo, Japan) under vacuum conditions.

### 3.11. Functional Properties of EAP

#### 3.11.1. Water-Holding Capacity

The water-holding capacity (WHC) of the EAP was determined according to the method developed by Lin et al. [50] and improved by Bayar et al. [51]. A sample of 500 mg of the extracted EAP was placed in a centrifuge tube and weighed (tube with polysaccharide). Then, 50 mL of distilled water was added, and the suspension was held at room temperature for 1 h with vortexing for 5 s every 15 min. After 20 min centrifugation at 5000× *g* using a refrigerated centrifuge Rotina 380R (Hettich-Heinze GmbH & Co. KG, Spenge, Germany), the upper phase was eliminated and the tube was tilted to a 45° angle on a filter paper, and then drained for 30 min. Water-holding capacity was determined as the weight of the contents of the tube after draining divided by the weight of the dried EAP, and expressed as the weight % of dried EAP.

#### 3.11.2. Oil Holding Capacity (OHC)

Oil holding capacity (OHC) was measured by the method of Lin et al. [50] with slight modifications. EAP (0.5 g) was placed in a centrifuge tube and weighed (tube with EAP). Soybean oil (10 mL) was added and the EAP/oil solution was then vortexed for 5 s every 15 min, and up to 1 h. Subsequently, the mixture was centrifuged at 5869× *g* for 20 min with the refrigerated centrifuge Rotina 380R. The upper phase was discarded and the centrifuge tube was tilted to a 45° angle on a filter paper, and then drained for 30 min. The EAP fat-binding capacity was determined as the weight of the tube content after draining divided by the weight of the dried EAP, and expressed as the weight % of dried EAP.

#### 3.11.3. Foaming Properties

Foam capacity (FC) and foam stability (FS) of EAP were determined according to the method described by Ben Romdhane et al. [52]. Ten milliliters of EAP solution at different concentrations (*w*/*v*) ranging from 0.5% to 5% were homogenized using a Moulinex R62 homogenizer (Organotechnie, La Courneuve, France) to incorporate air for 2 min at room temperature (20 ± 1 °C). Whipped samples were then immediately transferred into a graduated cylinder to record the volume of foam formed after expansion. The foam volume remaining up to 60 min was measured.

Foam expansion, expressed as the percentage of volume increased immediately after homogenization, was calculated according to Equation (1).

(1) $$FC(\%)=\frac{V_{T}-V_{0}}{V_{0}}100$$

*FS* was measured as the volume of foam remaining after 10 min, 20 min, 30 min, and 60 min, compared with the initial volume before whipping. The *FS* was determined according to Equation (2).

(2) $$FS(\%)=\frac{V_{T}-V_{0}}{V_{0}}100$$

where *V_T_* is the total volume after whipping (mL), *V*_0_ is the volume before whipping, and *V_t_* is the total volume after leaving at room temperature between 10 min and 60 min.

#### 3.11.4. Emulsification Properties

The emulsification properties of EAP were assessed according to the method described by Ben Romdhane et al. [23], with modifications. Different samples of 30 mL EAP suspensions at different concentrations (0.5%, 1%, 2%, 3%, 4%, and 5%; *w*/*v*) were mixed with 10 mL soybean oil and homogenized for 3 min using a Moulinex R62 homogenizer. Aliquots of the emulsion (50 µL) were taken from the bottom of the container immediately, and after 10 min, and diluted with 5 mL of 0.1% sodium dodecyl sulfate (SDS) solution. Subsequently, the absorbance of the diluted emulsions was measured at 500 nm using a spectrophotometer UV-mini 1240 (Shimadzu, Beijing, China). Emulsion capacity (EC) was determined by reading the absorbance immediately after the formation of the emulsion. The emulsion activity index (EAI) was then evaluated according to Equation (3).

(3) $$EAI(m^{2}\times g^{-1})=\frac{2\times 2.303\times A_{0}}{\varphi \times EAPweight(g)}$$

where *A*_0_ is the absorbance measured immediately after emulsion formation at 500 nm, and *Φ* presents the oil volumetric fraction (0.25).

The emulsion stability (ES) was evaluated according to Equation (4).

(4) $$ES(\%)=\frac{A_{10}}{A_{0}}100$$

where *A*_0_ and *A*_10_ correspond to the initial absorbance and that measured after 10 min, respectively.

### 3.12. Antioxidant Activity of EAP

#### 3.12.1. Determination of Total Antioxidant Capacity

The total antioxidant activity (TAC) of the EAP was measured by the phosphate molybdate test according to the method described by Bouaziz et al. [53]. The assay is based on the reduction of Mo (VI) to Mo (V) by the extract and subsequent formation of a green phosphate/Mo (V) complex at acidic pH. Samples (100 µL) at various concentrations (0.3, 05, 0.7, 1, 3, 5, 7, and 10 mg/mL) were mixed with 1 mL of reagent (0.6 M sulfuric acid, 28 mM sodium phosphate, and 4 mM ammonium molybdate), and the mixtures were incubated at 95 °C for 90 min. After cooling to room temperature, the absorbance was measured at 695 nm. BHA was used as a positive control, and the antioxidant activity was expressed as ascorbic acid equivalent using a standard curve.

#### 3.12.2. DPPH Radical-Scavenging Activity

The DPPH• radical scavenging activity of EAP was assessed using the method described by Bouaziz et al. [54]. A volume of 500 µL of each sample at different concentrations (0.2, 0.4, 0.6, 0.8, and 1 mg/mL) was mixed with 375 µL of ethanol (99%) and 125 µL of DPPH solution 0.02% (*w*/*v*) prepared in ethanol. The mixture was stirred and then incubated in the absence of light at room temperature for 1 h. The scavenging capacity was determined by monitoring the decrease of the absorbance at 517 nm. The DPPH radical had a maximum absorbance at 517 nm, which disappeared upon reduction by an antiradical compound. BHA was used as a positive control. The DPPH radical scavenging activity was calculated according to Equation (5).

(5) $$DPPH\;radical\;scavenging\;activity\;(\%)=\frac{A_{control}-A_{sample}}{A_{control}}100$$

where *A_control_* is the absorbance of the control reaction containing all the reagents except the sample, and *A_sample_* is the absorbance of the sample with the DPPH solution.

#### 3.12.3. ABTS Radical Scavenging Activity

The ABTS radical scavenging activity of EAP was determined according to the method described by Bayar et al. [51], with modifications. The ABTS radical cation was the result of the reaction between aqueous solutions of ABTS (2,2′-azino-bis(3-ethylbenzothiazoline-6-sulfonic acid)) (7 mM) and potassium persulfate (2.45 mM), after incubation for 12 h in obscurity at room temperature. The mixture was then diluted in ethanol to get an absorbance of 0.70 ± 0.02 at 734 nm. For this test, different concentrations (1–4 mg/mL) of EAP solutions were used and 0.5 mL samples were mixed with 1 mL ABTS radical. After 6 min incubation at room temperature in obscurity, the absorbance of the mixture was measured at 734 nm. Trolox was used as a positive control, and the ABTS radical scavenging activity was calculated with the same formula of the DPPH radical scavenging activity (Equation (5)).

#### 3.12.4. β-Carotene-Linoleic Acid Assay

The ability of EAP to prevent bleaching of β-carotene was determined as described by Koubaa et al. [55]. A stock solution of β-carotene/linoleic acid mixture was prepared by dissolving 0.5 mg of β-carotene, 25 µL of linoleic acid, and 200 µL of Tween 40 in 1 mL of chloroform. After evaporation of the chloroform under vacuum in a rotatory evaporator (Heidolph Instruments GmbH & Co. KG, Schwabach, Germany) at 40 °C, 100 mL of bi-distilled water was added, and the resulting mixture was vigorously stirred. The emulsion obtained was freshly prepared before each experiment. Aliquots (2.5 mL) of the β-carotene/linoleic acid emulsion were transferred to test tubes containing 0.5 mL of each EAP at different concentrations (1–5 mg/mL). After incubation for 2 h at 50 °C, the absorbance of each sample was measured at 470 nm. BHA was used as a positive standard. A control consisting of 0.5 mL distilled water was used instead of the sample. The antioxidant activities of EAP were evaluated in terms of β-carotene bleaching according to Equation (6).

(6) $$A(\%)=\left(1-\frac{A_{0}-A_{2}}{A_{0}{}_{0}-A_{02}}\right)\times 100$$

where *A*_0_ and *A*_2_ are the absorbance values measured in the presence of EAP at t = 0 and t = 2 h, respectively. *A*_00_ and *A*_02_ are the absorbance values measured in the absence of EAP at t = 0 and t = 2 h, respectively.

#### 3.12.5. Reducing Power Assay

The EAP ability to reduce iron (III) was evaluated as described by Ben Romdhane et al. [52]. A sample aliquot of 0.5 mL EAP at various concentrations (1–5 mg/mL) was mixed with 1 mL of 0.2 M phosphate buffer (pH 6.6) and 1 mL of 1% (*w*/*v*) K_3_Fe(CN)_6_ solution. After incubation at 50 °C for 20 min, the reaction was stopped by adding 1 mL of 10% (*w*/*v*) trichloroacetic acid (TCA) solution, and the mixture was centrifuged for 10 min at 2113× *g*. An aliquot (1.5 mL) from the upper phase was diluted with 1.5 mL of distilled water and 0.1 mL of 0.1% (*w*/*v*) FeCl_3_ solution. The absorbance values of the samples and ascorbic acid, used as a reference molecule, were measured at 700 nm using the UV-mini 1240 spectrophotometer after incubation for 10 min. The higher absorbance of the reaction mixture indicated a greater reducing power. A control tube was conducted in the same conditions, where distilled water was used instead of the sample.

#### 3.12.6. Ferrous Ion-Chelating Activity

The chelating antioxidant activity of EAP toward ferrous ions (Fe^2+^) was measured according to the method described by Carter [56]. The chelating ability was monitored by measuring the decrease in the red color complex (Fe^2+^-ferrozine) at 562 nm. EDTA was used as a standard antioxidant compound. The inhibition percentage of ferrozine-Fe^2+^ complex formation was calculated according to Equation (7).

(7) $$Ferrous\;ion-chelating\;activity\;(\%)=\frac{A_{control}+A_{blank}-A_{sample}}{A_{control}}100$$

where *A_control_* is the absorbance of the control (without EAP), *A_blank_* is the absorbance of the EAP (without ferrozine), and *A_sample_* is the absorbance of the reaction tubes (using EAP with the ferrozine as a mixture).

### 3.13. Determination of the ACE Inhibitory Activity of EAP

The ACE inhibitory activity was measured as described by Nakamura et al. [57]. A sample solution containing different concentrations (1–5 mg/mL) of EAP (80 µL) was mixed with 200 µL of 5 mM hippuryl-L-histidyl-L-leucine (HHL), and then pre-incubated for 3 min at 37 °C. EAP was prepared in 100 mM borate buffer (pH 8.3), containing 300 mM NaCl. The reactions were then initiated by adding 20 µL of 0.1 U/mL ACE from rabbit lung prepared in the same buffer. After incubation for 30 min at 37 °C, the enzyme reactions were stopped by adding 250 mL of 0.05 M HCl. The liberated hippuric acid (HA) was extracted with ethyl acetate (1.7 mL) and then evaporated at 90 °C for 10 min. The residue was dissolved in 1 mL of distilled water and the absorbance of the extract was measured at 228 nm. The ACE inhibition percentage was calculated according to Equation (8).

(8) $$ACE\;inhibition\;(\%)=\frac{B-A}{B-C}100$$

where *A* is the absorbance of HA obtained in the presence of ACE inhibitor, *B* is the absorbance of HA without ACE inhibitors (100 mM borate buffer pH 8.3 was used instead of EAP), and C is the absorbance of HA without ACE (corresponding to HHL autolysis in the course of the enzymatic assay). The IC_50_ value, defined as the concentration of EAP (mg/mL) required to inhibit 50% of ACE activity, was calculated for each sample using non-linear regression from a plot of ACE inhibition percentage versus sample concentrations.

### 3.14. Statistical Analyses

All experiments were conducted in triplicate and the average values with standard deviation errors were reported.

## 4. Conclusions

In this work, water-soluble polysaccharides (EAP) were extracted from *E. alata* stems and characterized to investigate their functional properties, as well as their antioxidant and antihypertensive activities. The monosaccharide composition of EAP showed that glucose was the most abundant sugar (43.1%), followed by galactose (36.4%), mannose (14.9%), arabinose (3.7%), and gluconic acid (1.7%). EAP showed interesting water-holding capacity, fat-binding ability, foaming and emulsion properties, antioxidant activity, and potential ACE inhibition activity. These results proved that the EAP could be incorporated into different food formulations to improve their biological and functional properties. Nevertheless, deeper analyses should be performed on EAP to confirm the activities found in this work in vivo, as well as evaluate the cytotoxicity of EAP and the feasible scalability of the proposed process.

## Figures and Tables

**Table 1:** Characterization of the extracted polysaccharides from *Ephedra alata* (EAP).

Compound	Content *
Ash	10.24 ± 0.24
Carbohydrates	73.24 ± 1.94
Proteins	5.68 ± 0.01
Lipids	1.09 ± 0.31
Uronic acid	6.82 ± 0.57
Moisture	2.78 ± 0.3

* Ash, carbohydrates, proteins, lipids, and uronic acid are expressed in % dry weight (g/100 g dry material). Moisture content is expressed in % fresh material (g/100 g fresh material).

**Table 2:** Mineral composition of EAP expressed in mg/100 g of dry matter.

Mineral	mg/100 g of Dry Matter
Ca	197.85 ± 7.24
Na	223.2 ± 11.02
K	108.5 ± 4.10
Zn	0.140 ± 0.11
Mg	63.39 ± 12.25
Mn	0.563 ± 0.08
Fe	0.067 ± 0.12
Cu	<0.001

**Table 3:** Monosaccharide composition of EAP determined by gas chromatography-mass spectrometry (GC-MS).

Monosaccharide	Peak Area %
Galactose	36.4 ± 0.24
Arabinose	3.7 ± 1.02
Glucose	43.1 ± 1.10
Mannose	14.9 ± 0.11
Gluconic acid	1.7 ± 1.25
